# Elevated representational similarity of voluntary action and inhibition in Tourette syndrome

**DOI:** 10.1093/braincomms/fcad224

**Published:** 2023-08-24

**Authors:** Charlotte L Rae, Petar Raykov, Eleanor M Ambridge, Lincoln J Colling, Cassandra D Gould van Praag, Samira Bouyagoub, Liliana Polanski, Dennis E O Larsson, Hugo D Critchley

**Affiliations:** School of Psychology, University of Sussex, Brighton BN1 9QH, UK; School of Psychology, University of Sussex, Brighton BN1 9QH, UK; School of Psychology, University of Sussex, Brighton BN1 9QH, UK; School of Psychology, University of Sussex, Brighton BN1 9QH, UK; Wellcome Centre for Integrative Neuroimaging, University of Oxford, Oxford OX3 7JX, UK; Department of Neuroscience, Brighton & Sussex Medical School, Brighton BN1 9RY, UK; Center for Lifespan Psychology, Max Planck Institute for Human Development, Berlin 14195, Germany; School of Psychology, University of Sussex, Brighton BN1 9QH, UK; Department of Neuroscience, Brighton & Sussex Medical School, Brighton BN1 9RY, UK; Department of Neuroscience, Brighton & Sussex Medical School, Brighton BN1 9RY, UK; Sussex Partnership NHS Foundation Trust, Worthing BN3 7HZ, UK

**Keywords:** basal ganglia, intentional inhibition, pre-supplementary motor area, Tourette syndrome, voluntary action

## Abstract

Many people with Tourette syndrome are able to volitionally suppress tics, under certain circumstances. To understand better the neural mechanisms that underlie this ability, we used functional magnetic resonance neuroimaging to track regional brain activity during performance of an intentional inhibition task. On some trials, Tourette syndrome and comparison participants internally chose to make or withhold a motor action (a button press), while on other trials, they followed ‘Go’ and ‘NoGo’ instructions to make or withhold the same action. Using representational similarity analysis, a functional magnetic resonance neuroimaging multivariate pattern analysis technique, we assessed how Tourette syndrome and comparison participants differed in neural activity when choosing to make or to withhold an action, relative to externally cued responses on Go and NoGo trials. Analyses were pre-registered, and the data and code are publicly available. We considered similarity of action representations within regions implicated as critical to motor action release or inhibition and to symptom expression in Tourette syndrome, namely the pre-supplementary motor area, inferior frontal gyrus, insula, caudate nucleus and primary motor cortex. Strikingly, in the Tourette syndrome compared to the comparison group, neural activity within the pre-supplementary motor area displayed greater representational similarity across all action types. Within the pre-supplementary motor area, there was lower response-specific differentiation of activity relating to action and inhibition plans and to internally chosen and externally cued actions, implicating the region as a functional nexus in the symptomatology of Tourette syndrome. Correspondingly, patients with Tourette syndrome may experience volitional tic suppression as an effortful and tiring process because, at the top of the putative motor decision hierarchy, activity within the population of neurons facilitating action is overly similar to activity within the population of neurons promoting inhibition. However, not all pre-supplementary motor area group differences survived correction for multiple comparisons. Group differences in representational similarity were also present in the primary motor cortex. Here, representations of internally chosen and externally cued inhibition were more differentiated in the Tourette syndrome group than in the comparison group, potentially a consequence of a weaker voluntary capacity earlier in the motor hierarchy to suppress actions proactively. Tic severity and premonitory sensations correlated with primary motor cortex and caudate nucleus representational similarity, but these effects did not survive correction for multiple comparisons. In summary, more rigid pre-supplementary motor area neural coding across action categories may constitute a central feature of Tourette syndrome, which can account for patients’ experience of ‘unvoluntary’ tics and effortful tic suppression.

## Teaser

On an intentional inhibition task, in which participants choose whether to move or not, participants with Tourette syndrome show greater representational similarity of action and inhibition. This occurs specifically in the pre-supplementary motor area, highlighting this region as a functional nexus of voluntary action control in tic disorders.

## Graphical Abstract

**Graphical Abstract:**
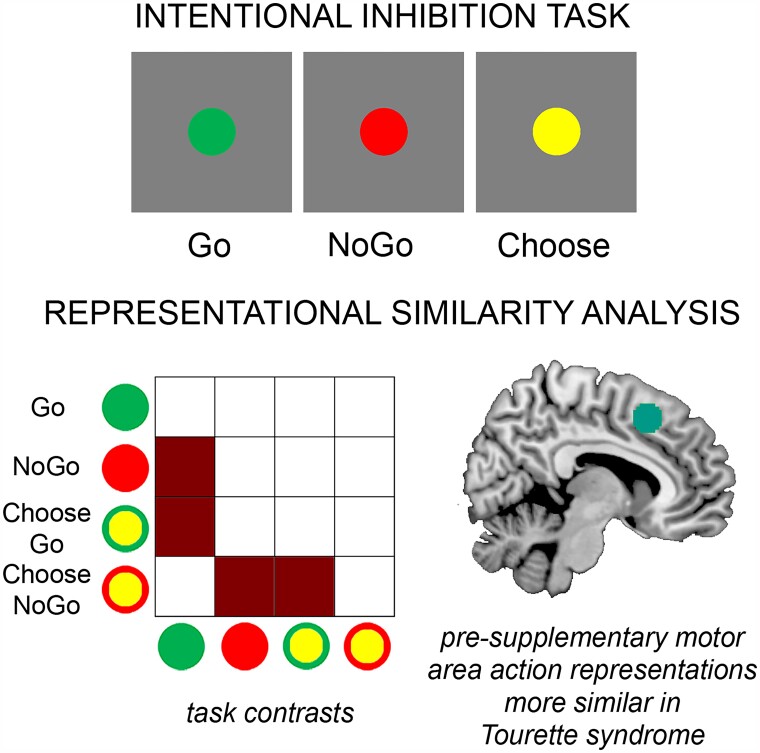

See Martino and Ganos (https://doi.org/10.1093/braincomms/fcad237) for a scientific commentary on this article.

## Introduction

Tourette syndrome (TS) is a neurodevelopmental hyperkinetic movement disorder, the primary feature of which is tics: rapid, recurrent, nonrhythmic movements and vocalizations. Tics are caused by aberrant interactions in cortico–striato–thalamo–cortical (CSTC) motor circuitry, likely through a combination of cortical hyperactivity, and direct pathway activation through the basal ganglia.^1,2^ Despite tics commonly being described as involuntary, many patients report feeling that their tics are a somewhat-voluntary response to an involuntary urge to move.^3^ Such premonitory urge sensations are experienced by the majority of adults with TS, and indeed this capacity of people with TS to attend to premonitory sensations forms the basis for targeted behavioural therapies.^4–6^

Another interesting feature of tics, also capitalized on by behavioural therapies, is that they are often suppressible.^7^ Many patients report using volitional tic suppression as a coping strategy in public settings where they face uncomfortable social scrutiny and stigma.^8,9^ This capacity to withhold tics volitionally is also considered when differentiating a TS diagnosis from other hyperkinetic movement disorders such as myoclonus.^7^ However, many people with TS report tic suppression to be a tiring and cognitively demanding process.

Neuroimaging studies suggest a basis for this effortful nature of tic suppression in TS: The inferior frontal gyrus (IFG) is hyperactive during volitional tic withholding, compared to ‘free ticcing’.^10^ Moreover, in people with TS, hyperactivity is observed in the right IFG during ‘intentional inhibition’ tasks, in which participants actively choose to withhold actions.^11^ The IFG is strongly associated with motor inhibition processes, including during the stop signal task, which tests externally cued stopping of motor output.^12,13^ During stopping, the IFG amplifies the neural drive from the pre-supplementary motor area (preSMA) to the subthalamic nucleus (STN), pausing motor outflow to the primary motor cortex (M1), via a route known as the hyperdirect pathway.^14,15^ Notably, during both externally cued and volitionally chosen motor inhibition, M1 activity is suppressed in non-TS individuals but is not in TS participants.^11^ This may explain why enhanced IFG activity is observed during tic suppression, since greater recruitment of inhibitory processes must compensate for downstream hyperactivity in M1.

A second subjective experience reported by patients is that during tic suppression, the tic (and premonitory urge) often does not ‘go away’ completely.^16^ This feature again implicates the hyperdirect pathway in pausing motor outflow by blocking the competing tic action plan in the direct pathway but not removing it.^1,17^ In order to exert this blocking effect, the IFG works together with the preSMA to amplify midline cortical drive to the STN, preventing motor output.^14^ Correspondingly, lesions to the preSMA disrupt stopping, slowing motor inhibition and predisposing to impulsive action.^18,19^

The preSMA has both a role in stopping action and as a cardinal site of voluntary action. Neurodegenerative conditions affecting the preSMA can engender either apathy at one end of the spectrum or alien limb phenomena and stimulus utilization behaviours at the other.^20,21^ Thus, clearly differentiated signals from preSMA down the motor hierarchy may enhance the efficient control of voluntary action, including decisions to move, decisions not to move and whether to act or inhibit responses to external cues from the environment. However, it remains unknown whether actions are represented differently within prefrontal regions, including preSMA and IFG, in the brains of people with TS. For example, are the prefrontal signals driving action and inhibition less clearly differentiated in TS? Is the lack of M1 suppression during cued inhibition a sign that representations of action and inhibition here are more similar? Is there also less differentiation of action and inhibition in TS within the basal ganglia—in particular at the striatal entry point to the direct and indirect pathways?

In addition to cortical and subcortical motor regions, increasing evidence suggests that the insular cortex interacts with downstream motor areas to cue tics in TS. This likely occurs via the generation of premonitory sensations, which then foster the production of tics in order to relieve the uncomfortable urges.^6,17,22^ How the insula might represent information relating to decisions to move, or to suppress movement, is speculative.

To understand better the neural mechanisms underlying tic suppression, we applied representational similarity analysis (RSA), a multivariate pattern analysis technique, to functional magnetic resonance neuroimaging (fMRI) data acquired as people with TS and comparison (non-TS) participants undertook an intentional inhibition task. In this paradigm, a modified Go/NoGo task, participants receive movement cues to either press a button (Go), withhold their button press (NoGo) or choose for themselves whether to press or inhibit (Choose).^23^ This task design enabled simultaneous investigation of voluntary action (on Choose trials when participants elect to press), intentional inhibition (on Choose trials when participants elect to withhold) and externally cued action (Go) and inhibition (NoGo). It also enabled direct comparison of action and inhibition processes between people with and without TS (while a tic suppression task can only be undertaken by patients).

Multivariate pattern analysis techniques can assess the granularity of neural activity within an area, to distinguish sub-populations of neurons associated with different task conditions.^24^ RSA is one multivariate technique, ideally suited to measuring the degree of similarity in neural responses to multiple stimuli classes or task conditions.^25^ In RSA, correlation coefficients across voxels are computed between conditions, such as externally cued or internally chosen action and inhibition.^26^

We hypothesized that people with TS would differ from non-TS individuals in the representation of action and inhibition and pre-registered our analyses. We examined six key regions previously implicated in motor action release or inhibition and to tic expression in TS: preSMA, IFG, bilateral insula, caudate nucleus and M1.^1^ Furthermore, we predicted that the similarity of action and inhibition neural representations in TS may relate to tic and premonitory sensation severity. Poorer discrimination between action and inhibition representations in TS may underpin the heightened cognitive effort experienced by many patients when attempting to suppress tics volitionally, through less clear-cut differentiation of action plans throughout CSTC circuitry. This lack of strongly differentiated higher-order motor action plans may worsen premonitory sensations and tic expression.

## Materials and methods

### Participants

The participants were identical to those described in a previous report^11^ (see for details of fMRI univariate analyses). Twenty-three individuals with TS (13 males; age 18–51, mean 34 years) and 21 individuals without TS and no history of major neurological or psychiatric disorder (11 males; age 19–55, mean 35 years) participated. Clinical diagnosis of TS was made by a UK neurologist or psychiatrist specialized in the assessment of TS. Patients were recruited from the Sussex Partnership NHS Foundation Trust (SPFT) Neurodevelopmental Service (psychiatrist H.D.C.) and Tourettes Action UK (specifying details of their clinical assessment prior to inclusion). Obsessive compulsive disorder (OCD) and attention deficit hyperactivity disorder (ADHD) diagnoses were also recorded.

Tic severity was assessed using the Yale Global Tic Severity Scale (YGTSS; symptom severity: maximum 50; impairment: maximum 50; global total: 100).^27^ fMRI analyses used the symptom severity score. Premonitory sensations were assessed using the Premonitory Urge for Tics Scale (PUTS);^28^ OCD severity using the Yale Brown Obsessive Compulsive Scale (YBOCS);^29^ and ADHD severity using the Adult ADHD Self-Report Scale (ASRS).^30^

Two patients were taking dopaminergic medications, six were taking serotonergic medications and one was taking both dopaminergic and serotonergic medications. One patient on sertraline also took a benzodiazepine. One patient took melatonin as a sleep aid remedy (although we did not class this individual as on medication in our statistical analyses, due to the distinct lack of psychoactive action relative to neurotransmitter medications). The remaining 13 patients and all participants in the comparison group were unmedicated.

Table 1 gives demographic details and clinical features (Supplementary Table 1: individual patient data). Participants gave written informed consent. The study was approved by the South East Coast: Brighton National Research Ethics Committee (15-LO-0109).

**Table 1:** Demographic details of participants, clinical features of patients and behavioural performance on the intentional inhibition task

Features/measures	Comparison (*n* = 21)	TS (*n* = 23)	Group difference
Number of males/females	11/10	13/10	*x* ^2^ = 0.439, *P* = 0.932
Age	35 (11)	34 (11)	*t* = 0.356, *P* = 0.724, BF_10_ = 0.313
Years of education	14 (2)	14 (2)	*t* = −0.010, *P* = 0.992, BF_10_ = 0.298
Number with OCD	0	10	
Number with ADHD	0	6	
YGTSS: symptom severity		26 (9)	
YGTSS: impairment		19 (13)	
YGTSS: total (symptom severity and impairment)		45 (19)	
PUTS		23 (7)	
ASRS	1 (1)	4 (2)	*t* = −4.474, ***P* < 0.001**, **BF_10_ = 351.15**
YBOCS	6 (6)	15 (10)	*t* = −3.457, ***P* < 0.001**, **BF_10_ = 25.70**
% Choose-Go	53% (10%)	56% (13%)	*t* = −0.924, *P* = 0.361, BF_10_ = 0.420
% NoGo errors	3% (3%)	3% (4%)	*t* = −0.228, *P* = 0.820, BF_10_ = 0.304
% Go omissions	1% (1%)	2% (2%)	*t* = −2.423, ***P* = 0.020**, BF_10_ = 2.920
Choose-Go reaction time (ms)	477 (45)	488 (43)	*t* = −0.887, *P* = 0.380, BF_10_ = 0.409
NoGo error reaction time (ms)	371 (182)	370 (166)	*t* = 0.018, *P* = 0.985, BF_10_ = 0.326
Go reaction time (ms)	419 (37)	434 (40)	*t* = −1.289, *P* = 0.204, BF_10_ = 0.579

Data are presented as means (SD). Group difference *P*-values refer to two-tailed *t*-tests or *χ*^2^ for number of males/females. Bold indicates *P* < 0.05 and/or BF_10_ > 3. OCD, obsessive compulsive disorder; ADHD, attention deficit hyperactivity disorder; YGTSS, Yale Global Tic Severity Scale; PUTS, Premonitory Urge for Tics Scale; YBOCS, Yale–Brown Obsessive Compulsive Scale; ASRS, Adult ADHD Self-Report Scale.

### Intentional inhibition task

**Figure 1:**
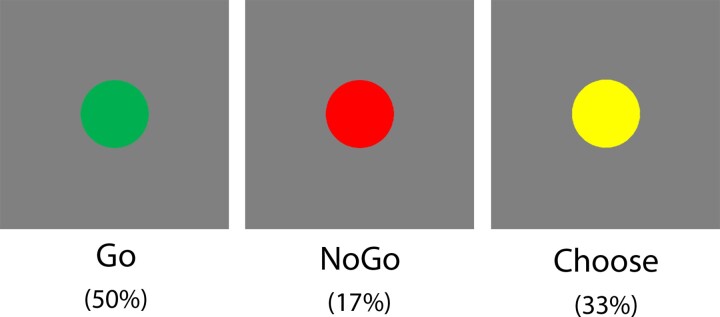
**Intentional inhibition task.** Following an intertrial interval, on Go trials (50%), green cues (left) instructed participants to make a button press; on NoGo trials (17%), red cues (middle) instructed participants to withhold; and on Choose trials (33%), yellow cues (right) indicated participants should choose whether to press or withhold. Stimuli enlarged for illustrative purposes. Re-printed from Rae, Parkinson, *et al.*^11^

Participants performed a modified Go/NoGo task in which movement cues (green, red and yellow circles) were presented on a grey background for 800 ms (Fig. 1; described in Rae, Parkinson, *et al.*^11^). Green Go cues indicated a button press to be made with the right index finger, red NoGo cues indicated the participant should withhold their button press and yellow ‘Choose’ cues indicated participants should choose whether to press the button or withhold. There were 864 trials: 432 Go (50%), 144 NoGo (17%) and 288 Choose (33%), presented in a pseudo-randomized order. The higher frequency of Go trials was designed to invoke a pre-potent tendency for action, as in traditional Go/NoGo tasks, to ensure that withholding on NoGo trials was sufficiently challenging and thus invoked inhibitory control.^23^ Participants were instructed to respond quickly on Go trials, withhold button presses on NoGo trials and choose quickly, making a fresh decision each time, on Choose trials.

A white fixation cross was displayed during intertrial intervals, jittered in duration and optimized using OptSeq (http://surfer.nmr.mgh.harvard.edu/optseq) for event-related design efficiency (35% 1000 ms, 30% 1130 ms, 20% 1250 ms, 10% 1380 ms and 5% 1500 ms). The task was divided into three runs of 288 trials, 10 min 42 s in duration, with breaks in between to reduce fatigue and discomfort.

### Intentional inhibition task statistical analysis

Motor behaviour (proportion of Choose trials when participants decided to act, i.e. %Choose-Go; NoGo commission errors; Go omissions; reaction times) was compared between TS and comparison participants (described in Rae, Parkinson, *et al.*^11^ and summarized here), using independent sample *t*-tests and Bayesian equivalents (applying default priors) in JASP (https://jasp-stats.org).

### MRI acquisition

T_2_*-weighted fMRI data were acquired on a Siemens Avanto 1.5T (32-channel head coil, repetition time = 2520 ms, echo time = 43 ms, 34 ascending 3 mm slices, 0.6 mm slice gap, in-plane resolution 3 × 3 mm). Two hundred and fifty-five fMRI volumes were acquired per 10 min run (765 volumes total). The first five volumes per run were discarded for steady-state magnetization. A T_1_-weighted image was acquired for fMRI pre-processing (repetition time = 2730 ms, echo time = 3.57 ms, 1 × 1 × 1 mm resolution). Participants’ heads were tightly cushioned within the head coil to reduce head movements.

### Tic monitoring

We did not instruct participants to suppress tics. This was essential to acquire intentional inhibition task fMRI data uncontaminated by simultaneous tic suppression in TS participants. Furthermore, not instructing participants to suppress tics reduced the likelihood of associated distress and fatigue over the imaging session. Instead, we removed fMRI signal relating to generation and expression of tics during the task. We video recorded tics, time locked to fMRI data, and included tic expression as a regressor in our general linear modelling (data and scripts available at https://osf.io/94ybj/). These videos, recorded concurrently with acquisition of neuroimaging data, enabled us to identify the timings of tics. This information was then used to exclude the effects of tic generation and expression from neuroimaging analyses but was not used to rate tic severity.

We acquired video data using both an in-bore MRI compatible camera (MRC Systems, www.mrc-systems.de), mounted on the head coil to view participants’ faces, and an out-of-bore camera to view limbs and body (360 × 240 resolution, 30 frames per second). Camera feeds and fMRI volume markers were simultaneously relayed to Spike2 physiological recording software (version 7.17, CED). During fMRI acquisition, the researcher (C.L.R.) watched the live video feeds and recorded the fMRI volumes at which she observed tics within a written record. This provided corroborative information and cover in case the video recordings were interrupted, lost or failed in another way. Storage of the video recording failed for three participants; in these cases, the written records alone identified tic onsets and durations in relation to the fMRI time series.

For the majority of participants with complete video recordings (*n* = 20), tics were identified in *post hoc* video assessment, using the written record as a supplementary guide. Initial tic ratings were conducted by two authors (L.P.: 8 videos; D.E.O.L.: 12), before a second rater, familiar with each patient’s tic repertoire (C.L.R.), conducted a second rating, confirming or rejecting the status of each event as a tic and identifying any tics not previously flagged by L.P. or D.E.O.L. An in-house Spike2 script extracted tic onsets and durations, time locked to fMRI data. Phonic tics were often visible from facial movement, but we did not record sound. This means it is possible that not every single tic was captured by our method; however, we believe it was as comprehensive as possible.

During the 30 min of fMRI, an average of 161 tics occurred (range 0–551; standard deviation: 147). The bodily locations at which tics were expressed were, on average, 40% facial, 8% head, 8% both face and head, 33% body or limbs and 11% combinations of face, head, body and limbs.

### fMRI pre-processing

fMRI data were pre-processed, and first-level general linear models fitted using SPM12 (v7219, www.fil.ion.ucl.ac.uk/spm; scripts available at https://osf.io/94ybj/). Pre-processing used default options, including realignment to the mean image, slice-time correction to the middle slice, co-registration with T_1_ structural and MNI normalization and 8 mm smoothing.

### First-level general linear modelling

A general linear model represented task events, with regressors for onset and duration (500 ms) of (i) Go, (ii) NoGo-correct, (iii) Choose-Go and (iv) Choose-NoGo trials. If participants made Go omissions or NoGo errors, regressors were added for these trial types. The general linear model of TS participants contained a further regressor for observed onsets and durations of tics. The fMRI data from the three runs were concatenated (spm_concatenate.m), adding a constant (mean) column for each of the three runs, and a ‘run transitions’ regressor modelled the transition from end of one run to the start of the next. Six realignment parameter regressors modelled head movement.

Single-regressor T-contrasts were generated for (i) Go, (ii) NoGo-correct, (iii) Choose-Go and (iv) Choose-NoGo trials, with implicit baseline of intertrial interval fixation cross. These were the key task conditions entered to RSA, the focus of this manuscript (see Rae, Parkinson, *et al.*^11^ for second-level statistical analysis and univariate brain activity results).

### Region of interest definition

**Figure 2:**
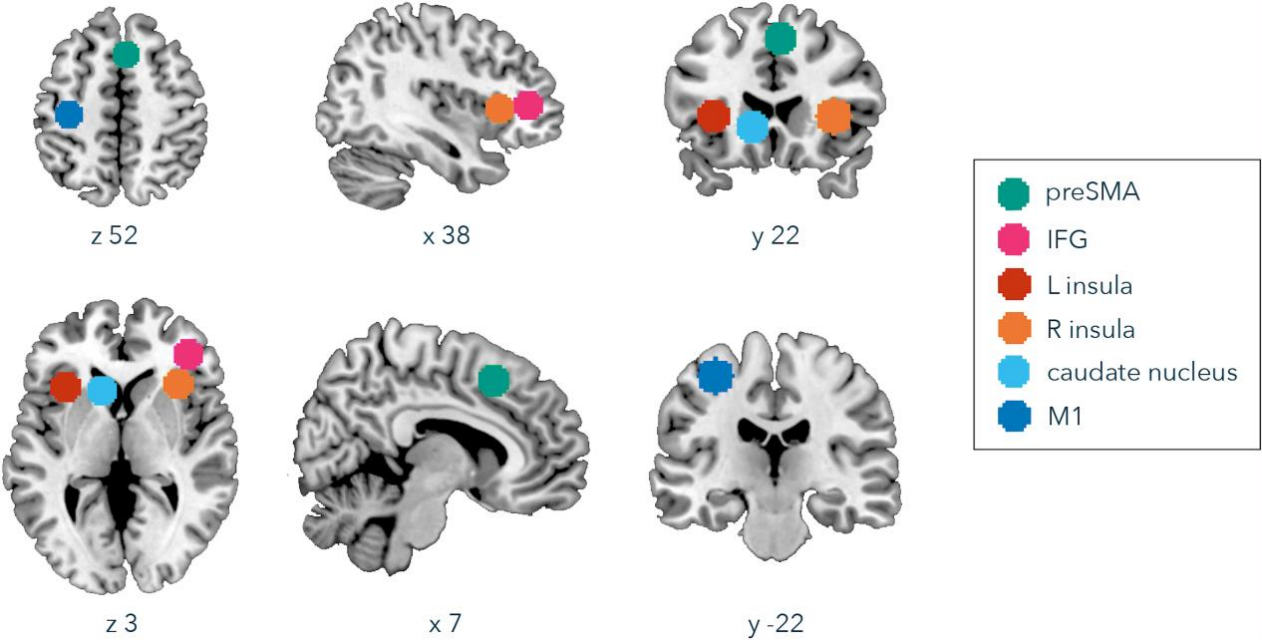
**ROIs entered to RSA.** Ten millimetre spheres centred on preSMA (green), IFG (pink), left insula (red), right insula (orange), caudate nucleus (light blue) and primary motor cortex (M1; dark blue) (see Table 2 for peak coordinates of each ROI sphere).

We focused analyses on six key regions previously identified as strongly associated with intentional inhibition task performance^11^ and that have also been identified as critical regions for symptom expression in TS^1,2^: the right preSMA, right IFG, bilateral insular cortex, left caudate nucleus and left primary motor cortex (Fig. 2). We defined our regions of interest (ROIs) by extracting a 10 mm sphere, centred on peak coordinates identified in the univariate analysis of Rae, Parkinson, *et al*.^11^ (Table 2), using MarsBaR (https://marsbar-toolbox.github.io/index.html). The masks were re-sliced (to an spmT image of one subject) using SPM co-registration, prior to application in RSA (mask files available at https://osf.io/6yknx/). We pre-registered the choice of these six ROIs prior to commencing RSA (https://osf.io/hx5ja/), but after data collection and fMRI univariate analysis, as the results in Rae, Parkinson, *et al*.^11^ informed ROI selection.

**Table 2:** Peak coordinates identified in the univariate analysis of Rae, Parkinson, *et al.*^11^ used to define ROIs for RSA (10 mm sphere centred on coordinate)

Region	Contrast used	*x*	*y*	*z*
preSMA (R)	F all effects	4	18	48
IFG (R)	Group difference conjunction	40	40	6
Insula (R)	F all effects	34	22	4
Insula (L)	F all effects	−34	20	4
Caudate nucleus (L)	Group difference NoGo and Choose-NoGo	−12	18	−2
M1 (L)	F all effects	−34	−22	56

### Contrast of interest definition

We focused our RSA comparisons on four key contrasts that enable investigation of externally cued and internally chosen action and inhibition: (i) Go versus NoGo, (ii) Choose-Go versus Choose-NoGo, (iii) Go versus Choose-Go and (iv) NoGo versus Choose-NoGo. In (i) and (iv), we enter only NoGo trials in which participants successfully withheld their button press (‘NoGo-correct’ in first-level general linear modelling above). The four relevant spmT images output from first-level models were merged into one 4D file for each subject, using the FSL command ‘fslmerge’. We pre-registered the choice of these four contrasts prior to commencing RSA (https://osf.io/hx5ja/), but after data collection and fMRI univariate analysis, as the results in Rae, Parkinson, *et al*.^11^ informed contrast selection. For some ROIs and contrasts (34%), we specified a directional hypothesis, predicting greater similarity in TS than non-TS participants (or vice versa), on the basis of prior literature (see https://osf.io/hx5ja/). For the remaining ROIs and contrasts (66%), we did not specify direction of effect, simply hypothesizing an exploratory group difference.

### Representational similarity analysis

RSA was conducted using the CosMoMVPA toolbox^31^ and custom scripts in MATLAB (data and scripts available at https://osf.io/6yknx/). The first-level contrasts (*t*-maps) reflecting activity during (i) Go, (ii) NoGo-correct, (iii) Choose-Go and (iv) Choose-NoGo trials were entered to RSA. We investigated the similarity between spatial patterns of fMRI activity using four different representational similarity analyses,^25^ testing the four contrasts outlined above. For all analyses, we computed the multivoxel spatial pattern similarity across pairs of trials using correlation. The Pearson correlation values were then Fisher transformed and weighted according to a contrast matrix that identified which task conditions were to be compared. A 4 × 4 contrast matrix (Go, NoGo, Choose-Go and Choose-NoGo) enabled the specific task conditions required for each contrast to be identified, applying a contrast weighting of (1) to the relevant row and column. Fisher-transformed similarity scores were then entered to statistical analysis.

### Statistical analysis

We tested group differences in representational similarity between TS and comparison participants using two-tailed independent sample *t*-tests and Bayesian equivalents (applying default priors) in JASP (https://jasp-stats.org). The JASP default priors are 0.707 (Cauchy) for a *t*-test; 1 (stretched beta prior width) for a correlation; and 1 (prior concentration) for a *χ*^2^ test. Because our four contrasts, tested across six ROIs, led to 24 independent statistical tests, we corrected *P*-values for multiple comparisons, across the six ROIs, per contrast, using false discovery rate (FDR)–adjusted *P*-values in MATLAB (code provided by Anderson Winkler, https://brainder.org/2011/09/05/fdr-corrected-fdr-adjusted-p-values/).

To investigate whether representational similarity is associated with symptom severity, we tested for two-tailed correlations between representational similarity and (i) tic severity, as measured by YGTSS (symptom severity score), and (ii) premonitory sensation severity, as measured by PUTS, for each of the four contrasts, in each of the six ROIs. Because this led to 48 independent statistical tests (24 per symptom score), we corrected *P*-values for multiple correlations, across the six ROIs, per contrast and per symptom score, again using FDR-adjusted *P*-values.

We pre-registered the choice of statistical tests, number of tests and FDR correction for multiple tests prior to RSA (https://osf.io/hx5ja/). However, the Bayes factors (BFs) were not originally pre-registered and were added *post hoc*, in order to give complementary insight into potential evidence for both the null (BF_10_ < 0.3) and alternative (BF_10_ > 3) hypothesis. The BFs should therefore be considered an exploratory approach. For each test, we reject the null hypothesis when the associated *P*-value is <0.05 and the BF_10_ > 3.

At the suggestion of a reviewer, we also report a set of control analyses to test if the expression of tics during the fMRI task had potentially impacted on our RSA results. Here, we examined two-tailed correlations (frequentist and Bayesian) between preSMA representational similarity and the number of tics expressed during scanning, across each of the four task contrasts. Furthermore, we tested if medication status interacted with RSA scores in the TS participants, by comparing preSMA representational similarity scores between medicated and unmedicated participants, for each of the four task contrasts, using (frequentist and Bayesian) two-tailed *t*-tests.

**Figure 3:**
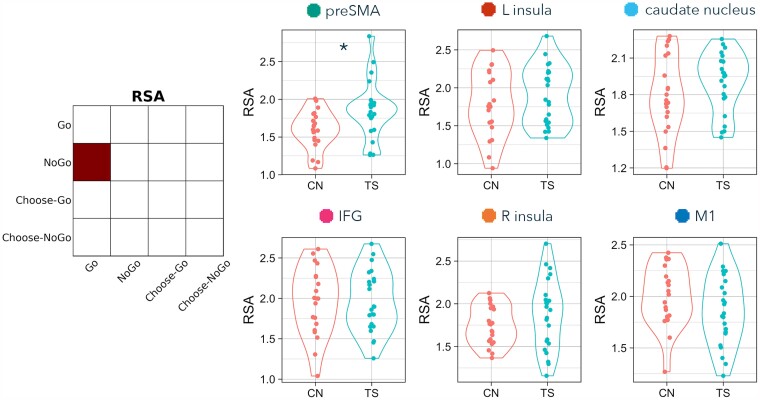
**RSA differences between TS and comparison (CN) groups on Go versus NoGo trials.** Contrast matrix illustrates RSA of Go versus NoGo. Plots present Fisher-transformed correlations between Go and NoGo trials in TS (blue) and CN (pink) participants. Statistical tests are two-tailed independent sample *t*-tests. An asterisk indicates both *P* < 0.05 and BF_10_ > 3.

**Figure 4:**
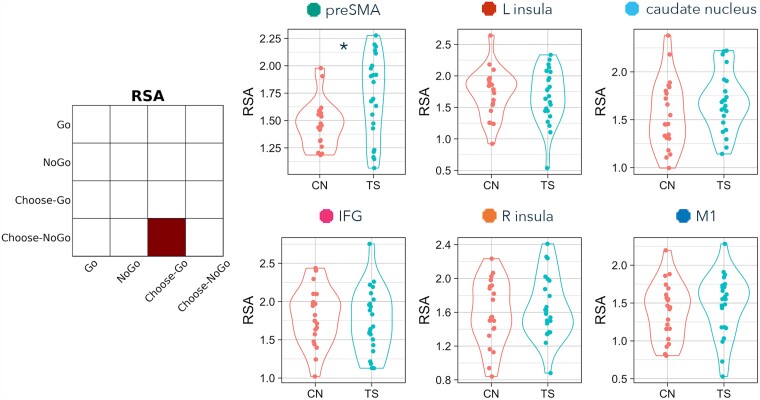
**RSA differences between TS and comparison (CN) groups on Choose-Go versus Choose-NoGo trials.** Contrast matrix illustrates RSA of Choose-Go versus Choose-NoGo. Plots present Fisher-transformed correlations between Choose-Go and Choose-NoGo trials in TS (blue) and CN (pink) participants. Statistical tests are two-tailed independent sample *t*-tests. An asterisk indicates both *P* < 0.05 and BF_10_ > 3.

**Figure 5:**
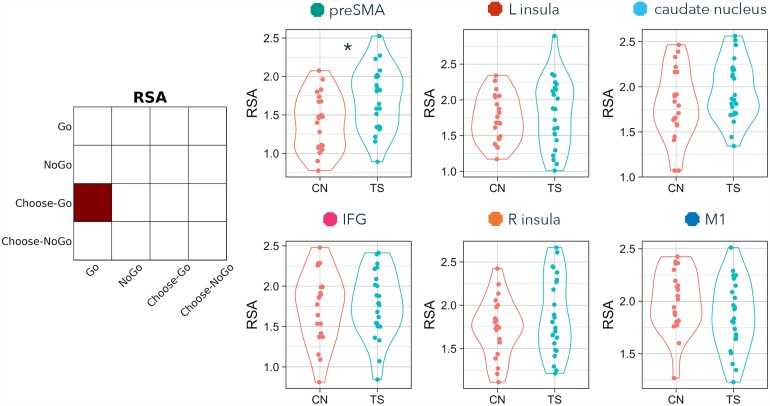
**RSA differences between TS and comparison (CN) groups on Go versus Choose-Go trials.** Contrast matrix illustrates RSA of Go versus Choose-Go. Plots present Fisher-transformed correlations between Go and Choose-Go trials in TS (blue) and CN (pink) participants. Statistical tests are two-tailed independent sample *t*-tests. An asterisk indicates both *P* < 0.05 and BF_10_ > 3.

**Figure 6:**
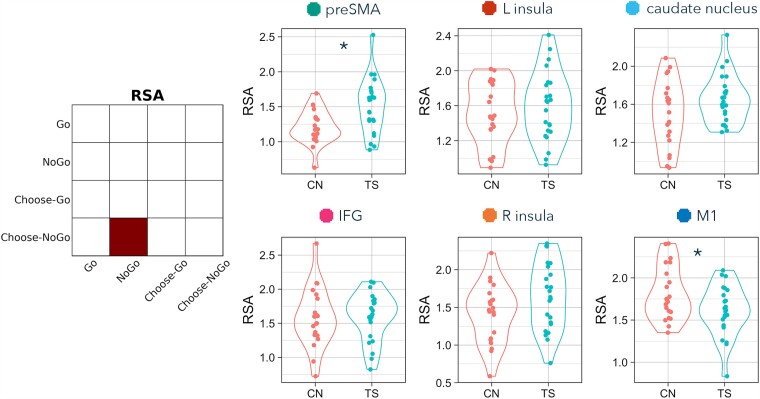
**RSA differences between TS and comparison (CN) groups on NoGo versus Choose-NoGo trials.** Contrast matrix illustrates RSA of NoGo versus Choose-NoGo. Plots present Fisher-transformed correlations between Go and Choose-Go trials in TS (blue) and CN (pink) participants. Statistical tests are two-tailed independent sample *t*-tests. An asterisk indicates both *P* < 0.05 and BF_10_ > 3.

Also at the suggestion of a reviewer, we explored the possibility that there are characteristic features of the sub-groups of TS participants that were potentially driving significant group differences by scoring beyond the range of comparison participants. To do so, we identified the TS individuals who scored above the preSMA RSA comparison maximum (i.e. those in the distribution plots in Figs 3–6 who can be seen to extend beyond the highest comparison RSA score). This allowed us to create two sub-groups of TS individuals: ‘greater’ (above the maximum comparison score) and ‘overlapping’ (equal to or less than the maximum comparison score). We then performed two-tailed *t*-tests and two-tailed *χ*^2^ tests (frequentist and Bayesian equivalents) to identify whether these two sub-groups of TS individuals were significantly different with regard to (i) tic severity (YGTSS), (ii) premonitory sensation severity (PUTS), (iii) frequency of tic expression, (iv) medication status, (v) ADHD diagnosis, and (vi) OCD diagnosis.

A third reviewer suggestion was to report in more detail the associations between behavioural performance on the intentional inhibition task and RSA scores. We tested for two-tailed correlations between preSMA RSA scores and six behavioural measures (%Choose-Go, %NoGo errors, %Go omissions, Choose-Go RT, NoGo RT and Go RT), across the four task contrasts. In addition, we took a similar ‘sub-group’ approach as outlined above, using two-tailed *t*-tests to identify whether the two sub-groups of TS individuals showed significantly different behavioural performance.

## Results

### Intentional inhibition task

TS participants did not choose to make or withhold actions (button presses) more often than the comparison group (%Choose-Go TS: 56%, comparison: 53%, *t* = −0.924, *P* = 0.361, BF_10_ = 0.420), nor did they make more NoGo errors (TS: 3%, comparison: 3%, *t* = −0.228, *P* = 0.820, BF_10_ = 0.304). TS participants made slightly more Go omissions (TS: 2%, comparison: 1%, *t* = −2.423, *P* = 0.020, BF_10_ = 2.920). Reaction times did not significantly differ between groups (Table 1).

### RSA: Go versus NoGo

The first RSA computed similarity of externally cued action (Go) and externally cued inhibition (NoGo-correct), for the six ROIs (Fig. 3 and Table 3). Representations of externally cued action and inhibition were more similar in TS than non-TS participants in the preSMA (*t* = −2.616, *P* = 0.012, BF_10_ = 4.185; although this group difference was no longer significant after correcting for multiple comparisons across the six pre-registered ROIs, pFDR = 0.074). Externally cued action and inhibition were not significantly different between TS and non-TS participants in any of the other ROIs (IFG: *t* = 0.063, *P* = 0.950, pFDR = 0.950, BF_10_ = 0.298; insula L: *t* = −0.878, *P* = 0.385, pFDR = 0.578, BF_10_ = 0.406; insula R: *t* = −1.249, *P* = 0.218, pFDR = 0.440, BF_10_ = 0.556; caudate nucleus: *t* = −1.244, *P* = 0.220, pFDR = 0.440, BF_10_ = 0.553; M1: *t* = 0.187, *P* = 0.852, pFDR = 0.950, BF_10_ = 0.302).

**Table 3:** Representational similarity of action and inhibition in non-TS comparison (CN) and TS participants, according to Fisher-transformed *Z*-scores (RSA) and raw correlation (CORR)

ROI	RSA		CORR		*P* (pFDR)	BF_10_
	CN	TS	CN	TS		
Go versus NoGo						
preSMA	1.596	1.853	0.912	0.940	**0.012** (0.074)	**4**.**185**
IFG	1.969	1.962	0.947	0.951	0.950 (0.950)	0.298
R insula	1.745	1.872	0.935	0.939	0.218 (0.440)	0.556
L insula	1.791	1.899	0.925	0.945	0.385 (0.578)	0.406
Caudate nucleus	1.794	1.902	0.935	0.951	0.220 (0.440)	0.553
M1	1.588	1.570	0.908	0.895	0.852 (0.950)	0.302
Choose-Go versus Choose-NoGo						
preSMA	1.461	1.715	0.891	0.920	**0.009** (0.057)	**5**.**141**
IFG	1.807	1.746	0.933	0.923	0.618 (0.648)	0.330
R insula	1.589	1.650	0.899	0.912	0.595 (0.648)	0.334
L insula	1.736	1.679	0.923	0.906	0.648 (0.648)	0.324
Caudate nucleus	1.558	1.689	0.898	0.923	0.198 (0.595)	0.590
M1	1.396	1.476	0.858	0.870	0.501 (0.648)	0.359
Go versus Choose-Go						
preSMA	1.402	1.714	0.861	0.917	**0.011** (0.068)	**4**.**445**
IFG	1.738	1.771	0.917	0.924	0.798 (0.798)	0.306
R insula	1.742	1.880	0.928	0.938	0.262 (0.393)	0.500
L insula	1.742	1.805	0.929	0.924	0.619 (0.743)	0.329
Caudate nucleus	1.804	1.957	0.932	0.953	0.169 (0.337)	0.652
M1	2.000	1.860	0.958	0.943	0.149 (0.337)	0.704
NoGo versus Choose-NoGo						
preSMA	1.209	1.510	0.823	0.884	**0.004** (**0.023**)	**10**.**466**
IFG	1.585	1.598	0.894	0.902	0.909 (0.910)	0.299
R insula	1.420	1.642	0.861	0.901	0.085 (0.145)	1.024
L insula	1.519	1.591	0.888	0.899	0.529 (0.635)	0.350
Caudate nucleus	1.500	1.655	0.886	0.922	0.097 (0.145)	0.934
M1	1.816	1.612	0.940	0.910	0.034 (0.102)	1.956

Statistics (*P*, pFDR, and BF_10_) are calculated on RSA values. Bold indicates *P* < 0.05 and/or BF_10_ > 3.

### RSA: Choose-Go versus Choose-NoGo

The second RSA computed similarity of internally chosen action (Choose-Go) and internally chosen inhibition (Choose-NoGo) (Fig. 4 and Table 3). Representations of chosen action and inhibition were more similar in TS than non-TS participants in the preSMA (*t* = −2.721, *P* = 0.009, BF_10_ = 5.141; although this group difference was no longer significant after correcting for multiple comparisons across the six pre-registered ROIs, pFDR = 0.057). Chosen action and inhibition were not significantly different between TS and non-TS participants in any of the other ROIs (IFG: *t* = 0.503, *P* = 0.618, pFDR = 0.648, BF_10_ = 0.330; insula L: *t* = 0.460, *P* = 0.648, pFDR = 0.648, BF_10_ = 0.324; insula R: *t* = −0.536, *P* = 0.595, pFDR = 0.648, BF_10_ = 0.334; caudate nucleus: *t* = −1.307, *P* = 0.198, pFDR = 0.595, BF_10_ = 0.590; M1: *t* = −0.679, *P* = 0.501, pFDR = 0.648, BF_10_ = 0.359).

### RSA: Go versus Choose-Go

The third RSA computed similarity of externally cued (Go) and internally chosen (Choose-Go) action (Fig. 5 and Table 3). Representations of externally cued and chosen action were more similar in TS than non-TS participants in the preSMA (*t* = −2.647, *P* = 0.011, BF_10_ = 4.445; although this group difference was no longer significant after correcting for multiple comparisons across the six pre-registered ROIs, pFDR = 0.068). Externally cued and chosen action were not significantly different between TS and non-TS participants in any of the other ROIs (IFG: *t* = −0.257, *P* = 0.798, pFDR = 0.798, BF_10_ = 0.306; insula L: *t* = −0.501, *P* = 0.619, pFDR = 0.743, BF_10_ = 0.329; insula R: *t* = −1.138, *P* = 0.262, pFDR = 0.393, BF_10_ = 0.500; caudate nucleus: *t* = −1.401, *P* = 0.169, pFDR = 0.337, BF_10_ = 0.652; M1: *t* = 1.470, *P* = 0.149, pFDR = 0.337, BF_10_ = 0.704).

### RSA: NoGo versus Choose-NoGo

The fourth RSA computed similarity of externally cued (NoGo) and internally chosen (Choose-NoGo) inhibition (Fig. 6 and Table 3). Representations of externally cued and chosen inhibition were more similar in TS than non-TS participants in the preSMA (*t* = −3.062, *P* = 0.004, BF_10_ = 10.466; and this group difference remained significant after correcting for multiple comparisons across the six pre-registered ROIs, pFDR = 0.023). In addition, there was a significant group difference in M1, in the opposite direction: representations of externally cued and chosen inhibition were more differentiated here in TS than in non-TS participants (*t* = 2.193, *P* = 0.034, BF_10_ = 1.956; however, this group difference was no longer significant after FDR correction across the six pre-registered ROIs, pFDR = 0.102). Externally cued and chosen inhibition were not significantly different between TS and non-TS participants in the other four ROIs (IFG: *t* = −0.114, *P* = 0.909, pFDR = 0.910, BF_10_ = 0.299; insula L: *t* = −0.634, *P* = 0.529, pFDR = 0.635, BF_10_ = 0.350; insula R: *t* = −1.766, *P* = 0.085, pFDR = 0.145, BF_10_ = 1.024; caudate nucleus: *t* = −1.697, *P* = 0.097, pFDR = 0.145, BF_10_ = 0.934).

### RSA: tic severity

**Figure 7:**
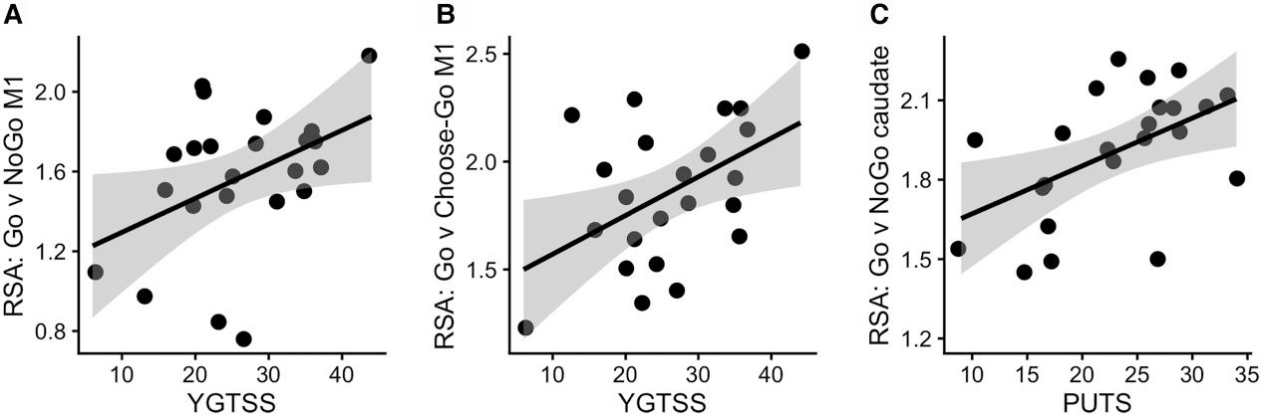
**RSA correlations with symptom severity in TS.** Two-tailed Pearson correlation tests. (**A**) Correlation of YGTSS with Go versus NoGo representational similarity (RSA) in primary motor cortex (M1) (*r* = 0.427, *P* = 0.042, BF_10_ = 1.817). (**B**) Correlation of YGTSS with Go versus Choose-Go RSA in M1 (*r* = 0.485, *P* = 0.019, BF_10_ = 3.447). (**C**) Correlation of PUTS with Go versus NoGo RSA in caudate nucleus (*r* = 0.513, *P* = 0.012, BF_10_ = 4.943). Grey interval around line of best fit = standard error of the mean. Each data point represents one TS participant.

In TS participants only, we tested for two-tailed correlations between similarity of representations in each of the four contrasts and tic severity, as measured by YGTSS (Table 4). This identified two significant associations between representational similarity and tic severity, both in M1 (Fig. 7A and B). The greater the representational similarity within M1 between externally cued action (Go) and inhibition (NoGo), the worse the tic severity (*r* = 0.427, *P* = 0.042, pFDR = 0.252, BF_10_ = 1.817), and the greater the similarity within M1 between externally cued (Go) and internally chosen (Choose-Go) action, the worse the tic severity (*r* = 0.485, *P* = 0.019, pFDR = 0.114, BF_10_ = 3.447). However, neither of these correlations remained significant after correcting for multiple tests across the six pre-registered ROIs.

**Table 4:** Correlations (Pearson’s *r*) between symptom severity (YGTSS and PUTS) and representational similarity of action and inhibition (according to Fisher-transformed *Z*-scores and RSA) in TS only

ROI	YGTSS			PUTS		
	*r*	*P* (pFDR)	BF_10_	*r*	*P* (pFDR)	BF_10_
Go versus NoGo						
preSMA	0.171	0.434 (0.532)	0.345	−0.141	0.521 (0.625)	0.314
IFG	0.168	0.443 (0.532)	0.341	0.105	0.635 (0.635)	0.288
R insula	−0.220	0.313 (0.532)	0.418	−0.192	0.380 (0.602)	0.372
L insula	−0.050	0.820 (0.820)	0.265	0.184	0.401 (0.602)	0.361
Caudate nucleus	0.205	0.348 (0.532)	0.392	0.513	**0.012** (0.072)	**4**.**943**
M1	0.427	**0.042** (0.252)	1.817	0.203	0.352 (0.602)	0.389
Choose-Go versus Choose-NoGo						
preSMA	0.007	0.973 (0.973)	0.259	−0.188	0.392 (0.755)	0.366
IFG	0.087	0.692 (0.973)	0.278	0.039	0.859 (0.877)	0.262
R insula	−0.145	0.510 (0.973)	0.317	−0.147	0.503 (0.755)	0.320
L insula	0.083	0.707 (0.973)	0.276	0.034	0.877 (0.877)	0.261
Caudate nucleus	0.007	0.973 (0.973)	0.259	0.181	0.408 (0.755)	0.357
M1	0.319	0.137 (0.822)	0.732	0.377	0.077 (0.462)	1.134
Go versus Choose-Go						
preSMA	0.251	0.248 (0.298)	0.486	0.109	0.620 (0.744)	0.290
IFG	0.309	0.151 (0.279)	0.683	−0.011	0.959 (0.959)	0.259
R insula	−0.040	0.856 (0.856)	0.263	−0.109	0.619 (0.744)	0.290
L insula	0.286	0.186 (0.279)	0.591	0.137	0.533 (0.744)	0.310
Caudate nucleus	0.367	0.085 (0.255)	1.046	0.167	0.447 (0.744)	0.339
M1	0.485	**0.019** (0.114)	**3**.**447**	0.367	0.085 (0.510)	1.049
NoGo versus Choose-NoGo						
preSMA	0.107	0.627 (0.752)	0.289	0.097	0.659 (0.791)	0.283
IFG	0.130	0.554 (0.752)	0.305	0.112	0.610 (0.791)	0.292
R insula	−0.234	0.282 (0.752)	0.446	−0.193	0.379 (0.758)	0.373
L insula	0.241	0.269 (0.752)	0.460	0.261	0.229 (0.687)	0.512
Caudate nucleus	−0.040	0.856 (0.856)	0.263	0.391	0.065 (0.390)	1.286
M1	0.142	0.518 (0.752)	0.315	0.008	0.973 (0.973)	0.259

Bold indicates *P* < 0.05 and/or BF_10_ > 3.

### RSA: premonitory sensation severity

In TS participants only, we tested for two-tailed correlations between similarity of representations in each of the four contrasts and premonitory sensation severity, as measured by PUTS (Table 4). This identified one significant association between representational similarity and premonitory sensation severity, located in the caudate nucleus (Fig. 7C). The greater the representational similarity within the caudate between externally cued action (Go) and inhibition (NoGo), the worse the premonitory sensation severity (*r* = 0.513, *P* = 0.012, pFDR = 0.072, BF_10_ = 4.943), although this was no longer significant after correcting for multiple tests across the six pre-registered ROIs.

### RSA: influence of tic expression and medication status

To examine if the propensity to tic frequently during the task affected RSA findings, we further tested for two-tailed correlations between preSMA representational similarity and the number of tics expressed during scanning, across each of the four task contrasts (reported at the suggestion of a reviewer, see Supplementary Table 2). For all four contrasts, there was no significant correlation between preSMA RSA scores and number of tics expressed, with all the BFs indicating evidence for the null (BF_10_ < 0.3). This suggests that frequency of tic expression did not relate to RSA scores.

Furthermore, we tested if preSMA representational similarity differed between medicated and unmedicated participants, for each of the four task contrasts (Supplementary Table 3). For three of the four contrasts, there was no significant difference. However, the difference in RSA scores from the Go-NoGo contrast approached significance (trending to be higher in the medicated than unmedicated group; *t* = 1.731, *P* = 0.053), although BFs for all four *t*-tests were inconclusive (between 0.3 and 3).

### Potential sub-group features

Also at the suggestion of a reviewer, we explored the possibility that sub-groups of TS participants, i.e. those with RSA scores greater than the maximum comparison group score, were potentially driving the significant preSMA group difference results. We compared ‘greater’ and ‘overlapping’ sub-groups of TS participants for differences in (i) tic severity (YGTSS), (ii) premonitory sensation severity (PUTS), (iii) frequency of tic expression, (iv) medication status, (v) ADHD diagnosis, and (vi) OCD diagnosis. None of the *t*-tests or *χ*^2^ tests (Supplementary Tables 4 and 5) were significant, and all the BF_10_ values were inconclusive (between 0.3 and 3), indicating that the data are insufficient to conclude whether the TS participants scoring above the comparison participant RSA maximum formally differ from those scoring equal to or less than the comparison maximum.

### Behavioural performance and RSA

We tested for two-tailed correlations between preSMA RSA scores and six behavioural measures (%Choose-Go, %NoGo errors, %Go omissions, Choose-Go RT, NoGo RT and Go RT), across the four task contrasts (Supplementary Table 6). Only one correlation was significant (%NoGo errors and Choose-Go versus Choose-NoGo; *r* = −0.421, *P* = 0.046), but this would not pass correction for multiple comparisons with FDR. Moreover, ∼40% of the BFs in this analysis suggested evidence for the null (BF_10_ < 0.3).

We undertook an additional ‘sub-group’ approach similar to that outlined above, using two-tailed *t*-tests to identify whether the two sub-groups of TS individuals scoring above or less than the comparison maximum showed significantly different behavioural performance (Supplementary Table 7). Only one comparison was significant (longer NoGo RT in greater than overlapping participants on Choose-Go versus Choose-NoGo preSMA RSA scores; *t* = 2.295; *P* = 0.035), but this would not pass correction for multiple comparisons with FDR, and all BFs in this analysis were inconclusive (between 0.3 and 3).

Collectively, these analyses indicate that there is no strong evidence for an association between behavioural performance and RSA results in TS participants.

## Discussion

We compared representational similarity of action and inhibition between people with and without TS, finding that across every task condition, the preSMA showed greater representational similarity in TS. Whether comparing externally cued action and inhibition, internally chosen action and inhibition, externally cued and chosen action or externally cued and chosen inhibition, multivariate representations were more similar in TS. This highlights the preSMA as a nexus of less clearly differentiated action and inhibition plans in TS. We also observed an interesting reversal of this pattern in M1, with more differentiated representation of externally cued and chosen inhibition here in TS than in non-TS participants. However, it is worth noting that not all group differences survived correction for multiple comparisons. Finally, we investigated whether representational similarity was associated with symptom severity: three correlations suggested that a patient’s tics and premonitory sensations are worse when there is greater similarity in M1 and in the caudate nucleus, between externally cued action and inhibition, and between externally cued and chosen action (although these correlations were no longer significant after correcting for multiple tests).

Overall, our findings suggest that while regions such as IFG and M1 may differ at the univariate level in general amount of activity, within preSMA, sub-populations of neurons show less clear-cut differentiation when coding for different action processes in TS. This blurring of action and inhibition within the preSMA of people with TS may explain, in part, the subjective experience of many patients that volitional tic suppression is a tiring and effortful process—because, at the top of the motor decision hierarchy, activity in the population of neurons signalling action is more similar to activity from the population of neurons signalling inhibition.

### Mechanisms of representational similarity

Our key finding that the preSMA showed elevated similarity of action representations in TS may arise through a number of mechanisms. Previously, we observed that the preSMA was differentially activated for different conditions (e.g. internally chosen versus externally cued), in both TS and comparison participants.^11^ However, RSA is insensitive to the overall average activity within a region, and thus, it is unlikely that these univariate activations are driving the similarity scores reported here.

Our results suggest that action plans are more poorly distinguished in individuals with TS. This could be due to the preSMA having ‘poor eyesight’, i.e. is less well able to differentiate between the different conditions. However, it is unclear what led to this poorer discrimination across conditions. One possibility is that non-TS individuals exhibit similar brain pattern activations to the TS participants, just with more added noise. Additionally, higher similarity scores in TS participants could be driven by a sub-population of voxels responding to task difficulty, rather than representing the action plans *per se*. However, this would still suggest that the difficulty should be similar across conditions. Overall, these results suggest that the way in which volitional and externally driven action and inhibition plans are represented are less distinct, in terms of sub-populations of voxels, in TS, regardless of overall levels of activity.

### The preSMA in voluntary action and inhibition

The preSMA is critical to choosing whether to act or not. The preSMA has been identified in fMRI meta-analysis as a substrate for ‘whether’ action decisions,^32,33^ and electrical stimulation here famously elicits the urge to move.^34^ Given the preSMA’s role in intentional action and inhibition, it almost certainly underpins a TS patient’s decision to release, or to withhold, a tic.^17^

The readiness potential is a preSMA-generated event-related potential associated with voluntary decisions to move. It can be explained by an accumulation of neural evidence to threshold, at which point one commits to move.^35^ Some evidence suggests that people with TS show readiness potentials prior to the release of tics,^36,37^ indicating, in line with many patients’ subjective reports, that (some) tics are ‘somewhat-voluntary’ responses to an involuntary premonitory urge to move. This has led some authors to describe tics as ‘unvoluntary’.^3^

We found that representations were more similar in the preSMA in TS for intentional action (Choose-Go) and intentional inhibition (Choose-NoGo). This less clear-cut differentiation of action plans to move, or to withhold, may lead to a noisier accumulation-to-threshold process, generating a subjective experience of ‘unvoluntariness’ during tics. Reduced differentiation of representations may also account for the subjective experience reported by many people with TS that volitional tic suppression is a tiring and cognitively demanding process. During motor inhibition, the preSMA works with the IFG to direct the basal ganglia to pause motor outflow.^14,15,38^ However, greater similarity of volitional action and inhibition signals in the preSMA, as observed here, may reduce the strength of signalling to basal ganglia during intentional tic suppression. As a result, the IFG compensates by amplifying its output, accounting for the IFG hyperactivity during intentional inhibition observed in our previous univariate analysis.^11^ We suggest that this IFG hyperactivity, compensating for the reduced granularity between intentional action and inhibition in the preSMA, underlies the feeling of cognitive effort reported by patients.

While most preSMA group differences did not survive correction for multiple comparisons, it is notable that, in addition to the comparison between volitional action (Choose-Go) and inhibition (Choose-NoGo), we also observed greater representational similarity in the preSMA for each of the other three pairs of response conditions examined, namely, externally cued action and inhibition (Go and NoGo), externally cued and chosen action (Go and Choose-Go) and externally cued and chosen inhibition (NoGo and Choose-NoGo). Thus, inflexible or homogeneous preSMA representations across movement categories may constitute a central feature of TS.

Externally cued inhibition, as measured by contrasting Go versus NoGo, is supported by engagement of preSMA and dorsolateral prefrontal cortex.^39,40^ Interestingly, unless there is comorbid ADHD, externally cued inhibition is not impaired in TS^41^, a finding replicated here, as TS participants made no more NoGo errors than non-TS individuals (with a BF in fact suggesting evidence for the null hypothesis of no group difference, at BF_10_ = 0.3). Greater representational similarity between Go and NoGo trials in the preSMA therefore may not have significant ramifications for successful performance of the NoGo task in TS. Arguably, lateral prefrontal cortex resources are more important for environmentally signalled response inhibition (and when constrained, are implicated in poorer NoGo task performance in TS with comorbid ADHD).^40,41^

Our final two contrasts (Go versus Choose-Go and NoGo versus Choose-NoGo) compared externally cued instructions with internally chosen action or inhibition. Here, the critical difference is an internal choice over whether to move or not versus the production or withholding of an action in response to an external cue (although we note that Choose trials are still not truly ‘freely willed’ in the sense of spontaneous action, since participants are cued to make a ‘whether’ decision to move or not). It is curious, therefore, that the preSMA, the cardinal site of voluntary action, differentiates less strongly between externally cued and internally chosen action and inhibition in TS. An explanation may be that TS patients have a developmental, chronic, sometimes lifelong, experience of tics as ‘unvoluntary’ actions that are partly cued by bodily associated premonitory urge sensations. Notably, we previously observed (in the same data set) that strength of connectivity between the preSMA and the basal ganglia (specifically the caudate nucleus, globus pallidus and thalamus) correlated with premonitory urge severity.^11^ Speculatively, the experience of urge-driven movements over many years may erode the sense of agency and volition over one’s actions in general, leading to the attrition of distinct voluntary action representations in the preSMA. Alternatively, less representational distinction between internally chosen and externally cued movement plans may be a defining characteristic of this developmental condition, rather than arising through acquired adaptive processes during the life course. The testing and replication of our RSA results in children or adolescents with TS will help resolve such questions.

### Representational similarity of the primary motor cortex

In addition to the preSMA, we observed one other group difference in representational similarity: Activity in M1 during externally cued and internally chosen inhibition was more similar in non-TS participants than TS. Potentially this is a consequence of aberrant preSMA representation, suggested above. However, this greater distinction in TS between inhibiting in response to environmental cues, versus chosen inhibition, may alternatively reflect many patients’ everyday practice of active tic suppression (in contrast to non-TS participants, who do not have such frequent opportunities to invoke volitional withholding of action). M1 may become tonically tuned to suppress activity levels in this volitional state, in contrast to the more reflexive process of inhibiting in response to external cues. Indeed, applying TMS to M1 during tic suppression reveals reduced corticospinal excitability in TS.^42^

Interestingly, it was in M1 that we also observed correlations between representational similarity and tic severity. Tic severity has previously been found to correlate with M1 structural and functional connectivity, highlighting a relationship between symptom expression and the integrity of this area.^43,44^ Varying developmental time courses in the plasticity of GABAergic interneurons within M1 may underpin this observation.^45,46^ We also observed an association between premonitory sensation severity and the similarity of representations within the caudate nucleus. Notably, all correlations were positive, such that more severe symptoms were linked to greater representational similarity. A lack of distinction between motor plans may increase the likelihood of tic expression, or, alternatively, the greater expression of tics may lead to the confusion of action plans. Unfortunately, correlational relationships cannot distinguish between these alternative accounts.

However, the BF for one correlation was inconclusive (BF_10_ < 3) which, combined with the overall relative lack of symptom severity correlations across several ROIs and comparisons, indicates that representational similarity of action and inhibition has comparatively weak bearing on variability in symptom expression. Our limited sample size may also have constrained our power to detect weak relationships between RSA findings and symptom severity. Nevertheless, our sample had high symptom heterogeneity (see Table 1), from less affected individuals to those with more severe symptoms, sufficient for our prior preSMA and IFG connectivity analyses to reveal associations with tic and premonitory urge severity.^11^ We therefore cautiously interpret the three symptom severity correlations observed here.

### Representational similarity of the insular cortex and basal ganglia

We investigated similarity of action and inhibition in the insular cortex, given its likely critical role in generating urges to move,^17,47^ and also the caudate nucleus, as the striatal entry point to the direct and indirect motor pathways.^1,48^ We did not observe group differences in insular representations, suggesting the processing of bodily feelings, including generation of premonitory urges, is less important to performance of motor tasks, unless decisions to act are strongly driven by such urges. Thus, while representations of action within the insula may not be fundamentally different in TS, it remains useful to investigate, using multivariate pattern analyses, if insula representations differ in TS during other tasks that closely tap into bodily feelings.

We also observed no evidence for differential representation of action and inhibition in the caudate nucleus. The ROI that we selected lies in the anterior caudate, based on the peak coordinate from our prior univariate analysis.^11^ The anterior caudate receives inputs from prefrontal cortex, in contrast to the dorsolateral putamen, which predominantly receives inputs from premotor cortex.^48^ Thus, our ROI is perhaps not optimized for capturing signals relating to the motor entry point to the basal ganglia. Alternatively, overall levels of striatal activity may determine execution or withholding of action, rather than finer-grained multivariate representations.^11^

### Therapeutic implications

Our overall conclusion that action representations are more similar in the preSMA suggests that this region could be a useful target for therapeutic interventions that enhance separation of action and inhibition signals. For example, medications, behavioural therapies and non-invasive brain stimulation techniques may all affect preSMA function. Of these, brain stimulation perhaps offers the most (anatomically) targeted approach. However, recent promising neurostimulation interventions are directed at SMA (rather than preSMA)^49^ or at motor-related oscillations using median nerve stimulation.^50^ Our results may therefore speak more to behavioural therapies, which facilitate tic suppression through habituation to premonitory urge sensations,^5^ since this likely involves a volitional control process supported by the preSMA.

### Study limitations

With six (pre-registered) ROIs examined across four contrasts, we conducted a number of independent statistical tests and therefore pre-registered a FDR approach to adjust for multiple comparisons. When applying this correction, several results were no longer significant, although it is compelling that the preSMA was so consistently identified as altered in representational similarity across contrasts. Bayesian statistics (BFs) provide complementary information, indicating the weight of evidence for either the null (BF_10_ < 0.3) or experimental (BF_10_ > 3) hypothesis, which we therefore presented alongside corrected and uncorrected *P*-values. Notably, all four preSMA group differences showed a BF_10_ > 3. We also pre-registered a set of directional hypotheses based upon prior literature; none of these showed a significant difference. The group differences that we did observe were in regions and contrasts that we did not specify an *a priori* direction for (TS > comparison or comparison > TS). Thus, we note the exploratory nature of these results.

Notably, group differences were seemingly driven by sub-groups of TS participants, who deviated from the non-TS participants more markedly than the rest of the group. RSA values in many TS individuals overlapped with values in the non-TS participants. This reflects the high patient heterogeneity that is characteristic of TS, although it is curious that there were relatively few correlations between RSA values and symptoms, suggesting that the group differences are not related to severity of TS *per se*. As with many neurophenotypic expressions, it appears that representational similarity occurs along a spectrum from ‘extreme non-TS’ to ‘extreme TS’, with overlap of both groups. At the suggestion of a reviewer, we presented more formal tests of whether TS participants with higher RSA scores showed distinct characteristic features (e.g. in tic or premonitory sensation severity; number of tics expressed during scanning; and medication and comorbidity status) that might drive the overall group differences in preSMA. All tests returned inconclusive BFs (between 0.3 and 3), indicating that the data are insufficient to conclude if TS participants scoring above the comparison participant RSA maximum differ (as a clinical sub-group) to those scoring equal to or less than the comparison maximum. Given the limited sample size, it will be valuable in future investigations to enlarge the cohort, with a potentially stratified sample, in order to characterize sub-groups in greater depth. In addition, it will be valuable to seek replication of our RSA results in children or adolescents, to ascertain the extent to which greater similarity scores are driven by longer life experience of urge-driven ‘unvoluntary’ tics in adults with TS.

It will also be valuable for future studies to assess the potential influence of medication on RSA scores. In an exploratory analysis, we saw that for one contrast (Go versus NoGo), patients taking psychoactive medications had higher preSMA RSA scores than unmedicated participants. As a canonical test of externally cued response inhibition, this contrast may relate most strongly to executive function, which is known to be impacted by noradrenergic and serotonergic medications.^51,52^ As mentioned above, larger samples are required to tease apart such potential effects of medication on RSA scores.

We adapted a Go/NoGo task, incorporating Choose trials on which participants decided whether to press the button or not.^23^ This factorial design gives maximum analytical potential to investigate contrasts amongst externally cued and internally chosen action and inhibition. In addition, this has the advantage of being a task that both TS and non-TS groups can do, enabling direct group comparison, which a tic suppression paradigm does not. However, our task has arguably lower ecological validity than other approaches to measuring intentional inhibition, such as withholding eye blinks, which capture an urge to blink akin to an urge to tic.^53^ However, successful trial numbers can vary between participants, and such tasks do not always include the ability to test response inhibition concurrently, as we do with NoGo trials. Nevertheless, it would be interesting to apply RSA to eye blink suppression fMRI data to examine how results replicate across paradigms. It would also be useful to apply RSA to other task-based fMRI studies of TS to understand how (dis)similarity of representations in other key areas such as insular cortex is altered.

## Conclusion

In TS, representations of action plans within the preSMA manifest heightened similarity across all four categories of externally cued and internally chosen action and inhibition. Our results suggest that in people with TS, signals from preSMA to subcortical nuclei to pause motor outflow are likely to be less well defined. This difference may explain why greater prefrontal activity is required in TS in order to withhold actions, such as those occurring during tic suppression, accounting for the subjective experience of many patients that the volitional withholding of tics is an effortful and cognitively demanding process.

## Supplementary Material

fcad224_Supplementary_Data

## Data Availability

An OSF repository contains de-identified demographic, clinical and behavioural data; task code; behavioural statistical analyses in JASP; tic regressor data and scripts; and pre-processing and first-level model scripts (first reported in Rae, Parkinson, *et al.*^11^; https://osf.io/94ybj/). A second, new, OSF repository contains the spmT statistic images, ROI mask files, RSA scripts, RSA statistical analyses in JASP and R code for graphical illustration (https://osf.io/6yknx/). Univariate statistical images (reported in Rae, Parkinson, *et al.*^11^) are in NeuroVault at https://neurovault.org/collections/9056. The RSA pre-registered analysis plan is detailed at https://osf.io/hx5ja/. An independent statistician checked the reproducibility of statistical analyses conducted in this paper. Their reproducibility report is at https://osf.io/dr35v/.
