# Supplementary material for: Elevated representational similarity of voluntary action and inhibition in Tourette syndrome
Source: Brain Commun. 2023 Aug 24;5(5):fcad224. doi: 10.1093/braincomms/fcad224 (PMC10497185; doi:10.1093/braincomms/fcad224)
Supplement: fcad224_Supplementary_Data [file fcad224_supplementary_data.docx]

**Elevated representational similarity of voluntary action and inhibition in Tourette syndrome**

**SUPPLEMENTARY INFORMATION**

Charlotte L Rae^1^*, Petar Raykov^1^, Eleanor M Ambridge^1^, Lincoln J Colling^1^, Cassandra D Gould van Praag^2^, Samira Bouyagoub^3^, Liliana Polanski^4^, Dennis E O Larsson^1,3^, Hugo D Critchley^3,5^

^1^ School of Psychology, University of Sussex, Brighton, UK

^2^ Wellcome Centre for Integrative Neuroimaging, University of Oxford, Oxford, UK

^3^ Department of Neuroscience, Brighton & Sussex Medical School, Brighton, UK

^4^ Center for Lifespan Psychology, Max Planck Institute for Human Development, Berlin, Germany

^5^ Sussex Partnership NHS Foundation Trust, Worthing, West Sussex, UK

**Supplementary Table 1.** TS participant clinical features and medications. OCD = obsessive compulsive disorder; ADHD = attention deficit hyperactivity disorder; YGTSS = Yale Global Tic Severity Scale; PUTS = Premonitory Urge for Tics Scale; YBOCS = Yale-Brown Obsessive Compulsive Scale; ASRS = Adult ADHD Self-Report Scale.

| **Patient** | **OCD (1 yes / 0 no)** | **ADHD (1 yes / 0 no)** | **YGTSS: symptom severity** | **YGTSS: impairment** | **YGTSS: total (symptom severity & impairment)** | **PUTS** | **YBOCS** | **ASRS** | **Medications (daily dose in mg)** |
| --- | --- | --- | --- | --- | --- | --- | --- | --- | --- |
| 1 | 0 | 0 | 13 | 0 | 13 | 26 | 8 | 5 | Citalopram (10) |
| 2 | 1 | 0 | 35 | 30 | 65 | 29 | 28 | 6 |  |
| 3 | 1 | 1 | 44 | 40 | 84 | 26 | 32 | 6 | Pimozide (6) |
| 4 | 1 | 0 | 36 | 20 | 56 | 31 | 15 | 2 | Sertraline (50) |
| 5 | 0 | 0 | 25 | 10 | 35 | 18 | 14 | 5 |  |
| 6 | 0 | 0 | 27 | 10 | 37 | 17 | 16 | 2 |  |
| 7 | 1 | 1 | 31 | 50 | 81 | 34 | 21 | 6 |  |
| 8 | 1 | 1 | 22 | 10 | 32 | 27 | 17 | 5 |  |
| 9 | 1 | 0 | 28 | 20 | 48 | 27 | 27 | 5 | Sertraline (200)  Lorazepam (200) |
| 10 | 0 | 0 | 35 | 10 | 45 | 15 | 18 | 6 |  |
| 11 | 1 | 1 | 21 | 20 | 41 | 22 | 18 | 6 | Escitalopram (20)  Pimozide (2) |
| 12 | 0 | 0 | 24 | 30 | 54 | 26 | 19 | 6 | Melatonin (6) |
| 13 | 1 | 1 | 20 | 30 | 50 | 28 | 5 | 1 |  |
| 14 | 0 | 0 | 21 | 0 | 21 | 23 | 4 | 1 |  |
| 15 | 0 | 0 | 6 | 0 | 6 | 9 | 0 | 2 | Quetiapine (200) |
| 16 | 0 | 0 | 37 | 10 | 47 | 21 | 8 | 1 |  |
| 17 | 0 | 0 | 17 | 20 | 37 | 17 | 0 | 2 |  |
| 18 | 0 | 0 | 23 | 20 | 43 | 23 | 21 | 4 | Fluoxetine (40) |
| 19 | 1 | 0 | 36 | 20 | 56 | 33 | 16 | 6 |  |
| 20 | 0 | 1 | 20 | 30 | 50 | 10 | 31 | 5 | Sertraline (100) |
| 21 | 0 | 0 | 29 | 20 | 49 | 16 | 8 | 1 | Citalopram (30) |
| 22 | 1 | 0 | 34 | 20 | 54 | 29 | 8 | 0 |  |
| 23 | 0 | 0 | 16 | 10 | 26 | 17 | 2 | 5 |  |
| TOTAL | 10/21 | 6/21 |  |  |  |  |  |  |  |
| MEAN |  |  | 26 | 19 | 45 | 23 | 15 | 4 |  |

**Supplementary Table 2.** Correlations (Pearson’s r) between number of tics expressed during fMRI scanning and representational similarity in the preSMA (according to Fisher transformed z-scores, RSA), in Tourette syndrome (TS) only. (**bold** = p<0.05 and/or BF<0.3 or BF>3; *underline* = trend p<0.1)

| **Contrast** | **r** | **p** | **BF_10_** |
| --- | --- | --- | --- |
| Go-NoGo | -0.035 | 0.876 | **0.262** |
| ChooseGo-ChooseNoGo | -0.076 | 0.731 | **0.273** |
| Go-ChooseGo | -0.088 | 0.690 | **0.279** |
| NoGo-ChooseNoGo | 0.103 | 0.641 | **0.286** |

**Supplementary Table 3.** T-tests comparing preSMA representational similarity between medicated (n=9) and unmedicated (n=14) TS participants. (**bold** = p<0.05 and/or BF_10_<0.3 or BF_10_>3; *underline* = trend p<0.1)

| **Contrast** | **Mean medicated** | **Mean unmedicated** | **t** | **p** | **BF_10_** |
| --- | --- | --- | --- | --- | --- |
| Go-NoGo | 2.043 | 1.731 | -2.050 | *0.053* | 1.607 |
| ChooseGo-ChooseNoGo | 1.766 | 1.682 | -0.514 | 0.612 | 0.424 |
| Go-ChooseGo | 1.626 | 1.770 | 0.818 | 0.423 | 0.489 |
| NoGo-ChooseNoGo | 1.553 | 1.482 | -0.414 | 0.683 | 0.410 |

**Supplementary Table 4.** Comparison of sub-groups within the TS sample whose preSMA RSA scores are either ‘greater’ (scoring above the maximum comparison score), or ‘overlapping’ (scoring equal to or less than the maximum comparison score). T-tests comparing 1) tic severity (YGTSS), premonitory sensation severity (PUTS), and number of tics expressed during fMRI task. (**bold** = p<0.05 and/or BF_10_<0.3 or BF_10_>3; *underline* = trend p<0.1)

| **Contrast** | **Mean greater** | **Mean overlapping** | **t** | **p** | **BF_10_** |
| --- | --- | --- | --- | --- | --- |
| **A** Go versus NoGo (n=4 greater; n=19 overlapping) | | | | | |
| Tic severity (YGTSS) | 28 | 26 | 0.516 | 0.611 | 0.494 |
| Premonitory sensation severity (PUTS) | 21 | 23 | -0.478 | 0.637 | 0.488 |
| Number of tics expressed during fMRI task | 172 | 159 | 0.158 | 0.876 | 0.457 |
| **B** Choose-Go versus Choose-NoGo (n=6 greater; n=17 overlapping) | | | | | |
| Tic severity (YGTSS) | 28 | 26 | 0.435 | 0.668 | 0.441 |
| Premonitory sensation severity (PUTS) | 23 | 23 | -0.047 | 0.963 | 0.414 |
| Number of tics expressed during fMRI task | 180 | 155 | 0.348 | 0.731 | 0.431 |
| **C** Go versus Choose-Go (n=4 greater; n=19 overlapping) | | | | | |
| Tic severity (YGTSS) | 25 | 26 | -0.258 | 0.799 | 0.464 |
| Premonitory sensation severity (PUTS) | 22 | 23 | -0.400 | 0.694 | 0.477 |
| Number of tics expressed during fMRI task | 189 | 155 | 0.405 | 0.690 | 0.478 |
| **D** NoGo versus Choose-NoGo (n=7 greater; n=16 overlapping) | | | | | |
| Tic severity (YGTSS) | 29 | 25 | 1.072 | 0.296 | 0.596 |
| Premonitory sensation severity (PUTS) | 22 | 23 | -0.481 | 0.636 | 0.435 |
| Number of tics expressed during fMRI task | 139 | 171 | -0.473 | 0.641 | 0.434 |

**Supplementary Table 5.** Comparison of sub-groups within the TS sample whose preSMA RSA scores are either ‘greater’ (scoring above the maximum comparison score), or ‘overlapping’ (scoring equal to or less than the maximum comparison score). Chi-squared tests comparing medication status (YES/NO), ADHD diagnosis (YES/NO), and OCD diagnosis (YES/NO). (**bold** = p<0.05 and/or BF_10_<0.3 or BF_10_>3; *underline* = trend p<0.1)

| **Contrast** | **Number greater** | **Number overlapping** | **x^2^** | **p** | **BF_10_** |
| --- | --- | --- | --- | --- | --- |
| **A** Go versus NoGo (n=4 greater; n=19 overlapping) | | | | | |
| Medication status | 3 (YES), 1 (NO) | 6 (YES), 13 (NO) | 2.616 | 0.106 | 1.807 |
| ADHD diagnosis | 1 (YES), 3 (NO) | 5 (YES), 14 (NO) | 0.003 | 0.957 | 0.521 |
| OCD diagnosis | 1 (YES), 3 (NO) | 9 (YES), 10 (NO) | 0.673 | 0.412 | 0.743 |
| **B** Choose-Go versus Choose-NoGo (n=6 greater; n=17 overlapping) | | | | | |
| Medication status | 2 (YES), 4 (NO) | 7 (YES), 10 (NO) | 0.115 | 0.735 | 0.534 |
| ADHD diagnosis | 1 (YES), 5 (NO) | 5 (YES), 12 (NO) | 0.374 | 0.541 | 0.518 |
| OCD diagnosis | 2 (YES), 4 (NO) | 8 (YES), 9 (NO) | 0.340 | 0.560 | 0.598 |
| **C** Go versus Choose-Go (n=4 greater; n=19 overlapping) | | | | | |
| Medication status | 2 (YES), 2 (NO) | 7 (YES), 12 (NO) | 0.240 | 0.624 | 0.649 |
| ADHD diagnosis | 1 (YES), 3 (NO) | 5 (YES), 14 (NO) | 0.003 | 0.957 | 0.521 |
| OCD diagnosis | 1 (YES), 3 (NO) | 9 (YES), 10 (NO) | 0.673 | 0.412 | 0.743 |
| **D** NoGo versus Choose-NoGo (n=7 greater; n=16 overlapping) | | | | | |
| Medication status | 3 (YES), 4 (NO) | 6 (YES), 10 (NO) | 0.059 | 0.809 | 0.515 |
| ADHD diagnosis | 1 (YES), 6 (NO) | 5 (YES), 11 (NO) | 0.727 | 0.394 | 0.583 |
| OCD diagnosis | 2 (YES), 5 (NO) | 8 (YES), 8 (NO) | 0.910 | 0.340 | 0.747 |

**Supplementary Table 6.** Correlations (Pearson’s r) between task behavioural measures and representational similarity in the preSMA, in Tourette syndrome (TS) only. (**bold** = p<0.05 and/or BF_10_<0.3 or BF_10_>3; *underline* = trend p<0.1)

| **Contrast** | **r** | **p** | **BF_10_** |
| --- | --- | --- | --- |
| **A** Go versus NoGo | | | |
| %Choose-Go | 0.211 | 0.333 | 0.402 |
| %NoGo errors | -0.153 | 0.486 | 0.325 |
| %Go omissions | -0.037 | 0.868 | **0.262** |
| Choose-Go RT | -0.171 | 0.435 | 0.345 |
| NoGo (incorrect) RT | -0.045 | 0.856 | **0.288** |
| Go RT | -0.076 | 0.730 | **0.274** |
| **B** Choose-Go versus Choose-NoGo | | | |
| %Choose-Go | 0.082 | 0.708 | **0.276** |
| %NoGo errors | -0.421 | **0.046** | 1.700 |
| %Go omissions | -0.068 | 0.757 | **0.270** |
| Choose-Go RT | 0.090 | 0.684 | **0.280** |
| NoGo (incorrect) RT | 0.314 | 0.191 | 0.630 |
| Go RT | -0.037 | 0.868 | **0.262** |
| **C** Go versus Choose-Go | | | |
| %Choose-Go | 0.394 | *0.063* | 1.320 |
| %NoGo errors | -0.067 | 0.760 | **0.270** |
| %Go omissions | 0.055 | 0.803 | **0.266** |
| Choose-Go RT | -0.266 | 0.221 | 0.524 |
| NoGo (incorrect) RT | 0.147 | 0.549 | 0.336 |
| Go RT | -0.140 | 0.525 | 0.313 |
| **D** NoGo versus Choose-NoGo | | | |
| %Choose-Go | -0.229 | 0.293 | 0.435 |
| %NoGo errors | -0.292 | 0.176 | 0.612 |
| %Go omissions | 0.059 | 0.790 | **0.267** |
| Choose-Go RT | 0.217 | 0.321 | 0.412 |
| NoGo (incorrect) RT | 0.454 | *0.051* | 1.673 |
| Go RT | 0.240 | 0.270 | 0.459 |

**Supplementary Table 7.** Comparison of sub-groups within the TS sample whose preSMA RSA scores are either ‘greater’ (scoring above the maximum comparison score), or ‘overlapping’ (scoring equal to or less than the maximum comparison score). T-tests comparing 6 behavioural measures from the intentional inhibition task. Reaction times expressed in milliseconds. (**bold** = p<0.05 and/or BF_10_<0.3 or BF_10_>3; *underline* = trend p<0.1)

| **Contrast** | **Mean greater** | **Mean overlapping** | **t** | **p** | **BF_10_** |
| --- | --- | --- | --- | --- | --- |
| **A** Go versus NoGo (n=4 greater; n=19 overlapping) | | | | | |
| %Choose-Go | 66% | 54% | 1.708 | 0.102 | 1.119 |
| %NoGo errors | 3% | 3% | -0.076 | 0.940 | 0.455 |
| %Go omissions | 2% | 2% | -0.501 | 0.622 | 0.491 |
| Choose-Go RT | 465 | 493 | -1.186 | 0.249 | 0.706 |
| NoGo (incorrect) RT | 368 | 365 | 0.050 | 0.961 | 0.460 |
| Go RT | 416 | 437 | -0.981 | 0.338 | 0.615 |
| **B** Choose-Go versus Choose-NoGo (n=6 greater; n=17 overlapping) | | | | | |
| %Choose-Go | 57% | 56% | 0.157 | 0.877 | 0.417 |
| %NoGo errors | 2% | 4% | -0.778 | 0.445 | 0.508 |
| %Go omissions | 3% | 2% | 0.265 | 0.793 | 0.424 |
| Choose-Go RT | 494 | 486 | 0.370 | 0.715 | 0.433 |
| NoGo (incorrect) RT | 430 | 336 | 2.295 | **0.035** | 2.177 |
| Go RT | 430 | 435 | -0.235 | 0.817 | 0.421 |
| **C** Go versus Choose-Go (n=4 greater; n=19 overlapping) | | | | | |
| %Choose-Go | 66% | 54% | 1.771 | *0.091* | 1.195 |
| %NoGo errors | 3% | 3% | -0.193 | 0.849 | 0.459 |
| %Go omissions | 1% | 3% | -1.150 | 0.263 | 0.687 |
| Choose-Go RT | 475 | 491 | -0.694 | 0.495 | 0.529 |
| NoGo (incorrect) RT | 372 | 364 | 0.153 | 0.880 | 0.464 |
| Go RT | 423 | 436 | -0.558 | 0.583 | 0.501 |
| **D** NoGo versus Choose-NoGo (n=7 greater; n=16 overlapping) | | | | | |
| %Choose-Go | 59% | 55% | 0.740 | 0.467 | 0.485 |
| %NoGo errors | 2% | 4% | -0.853 | 0.403 | 0.516 |
| %Go omissions | 2% | 2% | 0.180 | 0.859 | 0.406 |
| Choose-Go RT | 488 | 488 | 0.019 | 0.985 | 0.401 |
| NoGo (incorrect) RT | 402 | 345 | 1.352 | 0.194 | 0.763 |
| Go RT | 429 | 436 | -0.364 | 0.720 | 0.420 |
